# Supplementary material for: Gut Microbiota as a Potential Predictive Biomarker in Relapsing-Remitting Multiple Sclerosis
Source: Genes (Basel). 2022 May 23;13(5):930. doi: 10.3390/genes13050930 (PMC9140870; doi:10.3390/genes13050930)
Supplement: Supplementary file 1 [file genes-13-00930-s001.zip › genes-1725541-supplementary.pdf]

SUPPLEMENTARY MATERIALS

Supplementary Table S1. Microbiota composition at different taxonomic levels.

Supplementary Tables S1a, S1b and S1c show median and quartiles 25% (p25) and 75% (p75) values for all RRMS patients for each phylum, family and genus members identified in the microbiota analysis, respectively.

Supplementary Table S1a. Phylum

Stats	median	p25	p75
Firmicutes	58.70	54.94	65.62
Bacteroidetes	30.81	23.81	37.91
Actinobacteria	2.82	1.17	5.15
Proteobacteria	2.48	1.73	3.13
Verrucomicrobia	0.44	0.01	3.25
Synergistetes	0.02	0.00	0.07
Lentisphaerae	0.01	0.00	0.02
Euryarchaeota	0.01	0.00	0.18

Supplementary Table S1b. Family

Stats	median	p25	p75
*Ruminococcaceae**	24.31	18.29	32.38
Bacteroidaceae	15.89	12.24	21.18
*Lachnospiraceae**	15.87	13.31	17.40
Porphyromonadaceae	5.08	3.29	7.19
Prevotellaceae	3.29	0.01	8.53
Rikenellaceae	2.79	1.97	3.24
Eubacteriaceae	2.56	1.63	3.81
Oscillospiraceae	2.43	1.59	4.04
Veillonellaceae	1.84	0.02	4.33
Erysipelotrichaceae	1.75	1.32	3.32
Acidaminococcaceae	1.19	0.00	1.99
Bifidobacteriaceae	1.04	0.61	3.09
Sutterellaceae	1.00	0.40	1.34
Coriobacteriaceae	0.64	0.35	1.77
Desulfovibrionaceae	0.55	0.40	1.06
Akkermansiaceae	0.44	0.01	3.25
Clostridiaceae	0.38	0.20	0.82
Streptococcaceae	0.19	0.09	0.44
Christensenellaceae	0.15	0.05	0.56
Defluviitaleaceae	0.12	0.02	1.30
Lactobacillaceae	0.10	0.00	0.49
Eggerthellaceae	0.09	0.05	0.17
Gracilibacteraceae	0.05	0.00	0.46
Enterobacteriaceae	0.02	0.00	0.13

Supplementary Table S1c. Genus

**Stats**	**median**	**p25**	**p75**
Bacteroides	15.89	12.24	21.18
Faecalibacterium	9.74	8.32	12.17
*Ruminococcus**	4.34	2.76	7.68
*Gemmiger**	3.33	2.55	5.67
Roseburia	3.17	1.48	4.99
Alistipes	2.79	1.97	3.24
Eubacterium	2.56	1.62	3.80
Oscillibacter	2.43	1.59	4.04
*Lachnoclostridium**	2.00	1.43	2.81
Barnesiella	1.97	0.70	2.40
Parabacteroides	1.96	1.56	3.29
Ruminiclostridium	1.82	1.13	3.58
Blautia	1.73	1.37	2.35
Phascolarctobacterium	1.12	0.00	1.99
Erysipelatoclostridium	1.10	0.36	1.75
Bifidobacterium	1.04	0.61	3.09
Fusicatenibacter	0.98	0.50	1.67
Intestinimonas	0.70	0.30	1.15
Dorea	0.58	0.38	0.71
Desulfovibrio	0.55	0.40	1.06
Collinsella	0.54	0.25	1.77
Sporobacter	0.52	0.26	1.05
Coprococcus	0.49	0.36	0.89
Akkermansia	0.44	0.01	3.25
Tyzzerella	0.33	0.22	0.67
Pseudoflavonifractor	0.31	0.13	0.45
Acetanaerobacterium	0.30	0.04	0.64
Odoribacter	0.27	0.11	0.66
Anaerobacterium	0.19	0.01	0.90
*Streptococcus**	0.19	0.09	0.42
Sutterella	0.18	0.00	0.48
Christensenella	0.15	0.05	0.56
Vallitalea	0.10	0.02	1.23
Lactobacillus	0.10	0.00	0.49
Lachnospira	0.10	0.01	0.52
*Prevotella**	0.09	0.01	8.53
Parasutterella	0.08	0.01	1.16
Anaerostipes	0.08	0.05	0.38
Dialister	0.07	0.00	3.16
Eisenbergiella	0.06	0.01	0.11
Gracilibacter	0.05	0.00	0.46
Hungatella	0.05	0.03	0.15
Clostridium	0.03	0.01	0.13
Lachnobacterium	0.02	0.00	0.02
Escherichia	0.01	0.00	0.06
Paraprevotella	0.00	0.00	0.27

Supplementary Figure S1. Analysis of cases and controls. Genera Ezakiella and Bilophila. Number of cases and controls with detection of genera sequences and mean log value of sequences for both genera are included.

Ezakiella

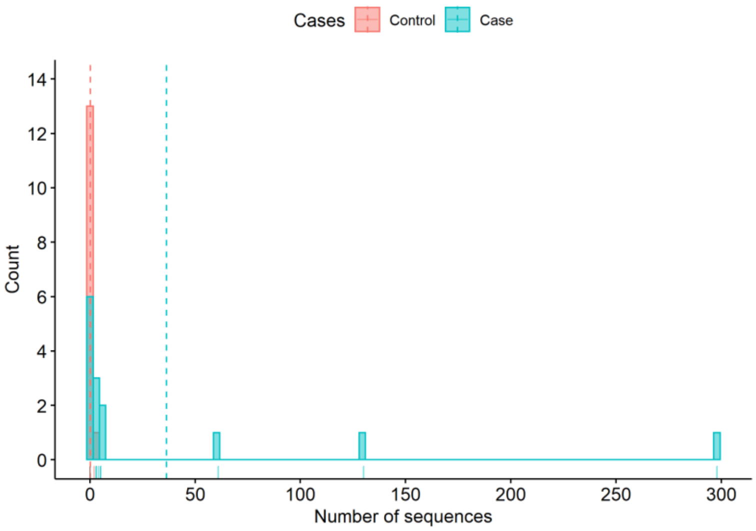

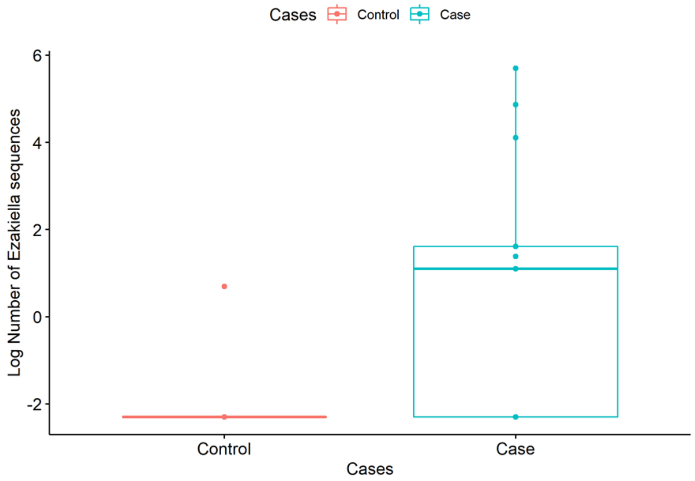

Bilophila

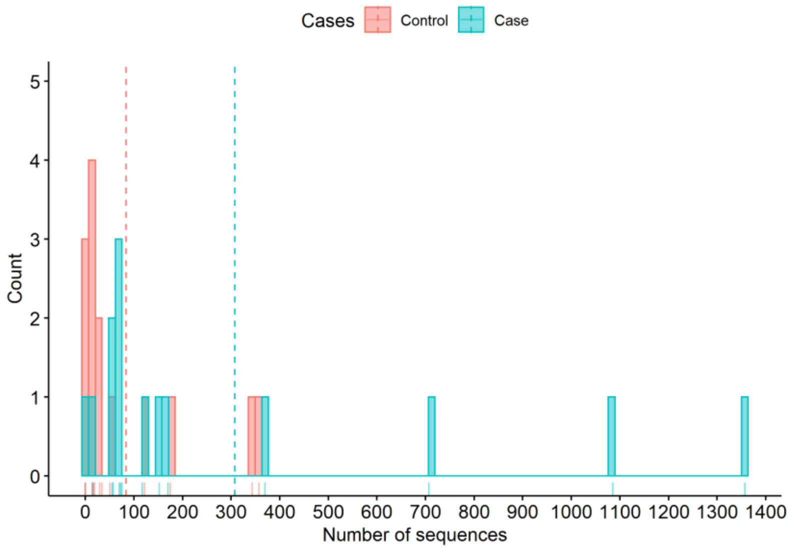
Cases
Control
Case
Count
0
1
2
3
4
5
0 100 200 300 400 500 600 700 800 900 1000 1100 1200 1300 1400
Number of sequences

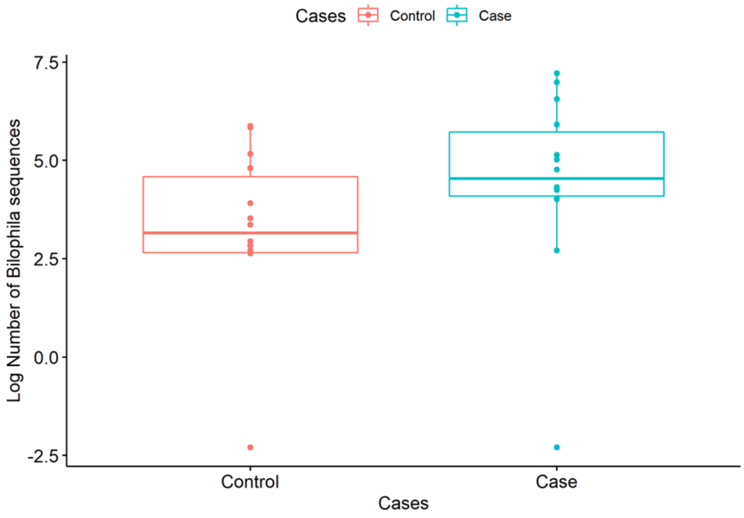
Cases
Control
Case
Log Number of Bilophila sequences
7.5
5.0
2.5
0.0
-2.5
Control
Case
Cases
